# Danshen protects against early-stage alcoholic liver disease in mice via inducing PPARα activation and subsequent 4-HNE degradation

**DOI:** 10.1371/journal.pone.0186357

**Published:** 2017-10-11

**Authors:** Lei Ding, Like Wo, Zhongyan Du, Lihua Tang, Zhenyuan Song, Xiaobing Dou

**Affiliations:** 1 College of Life Science, Zhejiang Chinese Medical University, Hangzhou, Zhejiang, P. R. China; 2 Zhejiang Provincial Hospital of Traditional Chinese Medical, Hangzhou, P. R. China; 3 Zhejiang Chinese Medical University, Hangzhou, Zhejiang, P. R. China; 4 Department of Kinesiology and Nutrition, University of Illinois at Chicago, Chicago, Illinois, United States of America; University of Louisville School of Medicine, UNITED STATES

## Abstract

Alcoholic liver disease (ALD) is a type of chronic liver disease caused by long-term heavy ethanol consumption. Danshen is one of the most commonly used substances in traditional Chinese medicine and has been widely used for the treatment of various diseases, and most frequently, the ALD. The current study aims to determine the potential beneficial effect of Danshen administration on ALD and to clarify the underlying molecular mechanisms. Danshen administration improved liver pathologies of ALD, attenuated alcohol-induced increment of hepatic 4-Hydroxynonenal (4-HNE) formation, and prevented hepatic Peroxisome proliferators activated receptor alpha (PPARα) suppression in response to chronic alcohol consumption. Cell culture studies revealed that both hepatoprotective effect and increased intracellular 4-HNE clearance instigated by Danshen supplementation are PPARα-dependent. In conclusion, Danshen administration can protect against ALD via inducing PPARα activation and subsequent 4-HNE degradation.

## Introduction

Alcoholic liver disease (ALD) is a type of chronic liver diseases caused by long-term heavy ethanol consumption. It has become a major public health problem worldwide and is a serious threat to human life. However, there is currently no safe and effective FDA-approved medications for ALD. Therefore, it is becoming urgent to discover efficient treatments to prevent and cure the disease in modern medicine.

The disease process of ALD is characterized by early steatosis (triglyceride accumulation in hepatocytes), steatohepatitis (steatosis with inflammatory cells infiltration and necrosis), with some individuals ultimately progressing to fibrosis. The cellular/molecular mechanisms underlying the development of early-stage pathologies of ALD (fatty liver and liver damage) are complex and considered to be multifactorial. Generally speaking, the development of hepatic steatosis results from an imbalance between triglyceride synthesis, which could result from increased fatty acids delivery from adipose tissue and hepatic de novo lipogenesis, and its cellular disposal, including decreased hepatic β-oxidation of fatty acids and inhibition of VLDL-triglyceride export. The effects of chronic alcohol consumption on all these metabolic pathways have been reported [1–4]. Peroxisome proliferators activated receptor alpha (PPARα), a member of the PPAR family of nuclear receptors, plays a critical role in regulating many genes involved in fatty acid oxidation in the liver [5–8]. Chronic alcohol consumption was associated with decreased PPARα expression and transactivity in the liver. Importantly, PPARα knockout exacerbated alcohol-induced fatty liver and liver injury [9], while PPARα agonist administration improved liver pathologies in mice induced by chronic alcohol exposure [2], suggesting that PPARα inhibition is a key mechanism during the development of ALD.

Hepatocyte cell death is one of major features in alcoholic hepatitis, the progressive form of ALD. The mechanism(s) underlying alcohol-triggered hepatocyte cell death remain to be fully elucidated. Among all proposed mechanisms, both increased production of proinflammatory cytokine, in specific TNF-alpha, and oxidative stress seem to play predominant roles in triggering hepatocyte cell death [10–14]. 4-Hydroxynonenal (4-HNE) is one of the most abundant and active lipid peroxides as a result of oxidative stress. The concentrations of 4-HNE in the liver and plasma were reported to be closely related to the development of ALD in both clinical and experimental settings [15–19] and a variety of membranous and cytoplasmic proteins modified by 4-HNE have been detected, which may mechanistically contribute to alcohol-induced hepatocyte dysfunction [20–21]. We previously reported that 4-HNE plays a pathological role in the development and progression of ALD via inhibiting hepatocyte nuclear factor-kappa-binding (NF-ΚB) activation, sensitizing hepatocytes to tumor necrosis factor-alpha (TNF-α) hepatotoxicity [22].

Danshen, the dried root of Salvia miltiorrhiza Bunge, is one of the most commonly used substances in traditional Chinese medicine and has been widely used for the treatment of various diseases, and most frequently, the ALD [23–25], mainly based on its antioxidant properties. Moreover, the liposoluble active ingredient of Danshen also reduced the liver injury induced by alcohol metabolites [26]. Interestingly, recent studies demonstrated that Danshen is capable of increasing PPARα expression in the tissue of rat liver and reducing the accumulated of lipid in the liver [27–31]. These previous findings altogether provided rational that Danshen can be a safe and efficient therapeutic choice for ALD. We hypothesize that Danshen injection can protect against ALD in mice via promoting hepatic fatty acid metabolism and reducing liver 4-HNE accumulation. In the present study, both in vivo animal studies and in vitro cell culture investigations were conducted to test our hypothesis.

## Materials and methods

### Ethics statement

This study did not involve non-human primates. All experiments described in this study were performed in full accordance with the guidelines for animal experiments released by the National Institute of Animal Health. This study is approved by the Animal Ethic Committee at Zhejiang Chinese Medicine University (ETHICS CODE Permit NO.SCXK (Zhe) 2008–0036).

### Chemicals

Danshen injection was purchased from Chiatai qingchunbao pharmaceutical Co. Limited (Hangzhou, China). Beta-Actin antibody and PPARα antibody were purchased from Santa Cruz Biotechnology (Dallas, Texas). 4-NHE antibody was purchased from R&D (Minnesota, US). Triglycerides assay kit and ALT assay kit were purchased from Nanjing Jiancheng Bioengineering Institute (Nanjing, China). LDH assay kit was purchased from Thermo scientific (Middletown, VA). 4HNE reagent was purchased from Cayman Chemical (Ann Arbor, Michigan). Other chemicals were obtained from Sigma-Aldrich (St.Louis, MO).

### Cell lines and culture conditions

The human hepatoma cell line HepG2 and the non-hepatoma hepatocyte cell line NCTC1469 were both obtained from the Shanghai Institute of Cell Bank. HepG2 were cultured in Dulbecco’s Modified Eagle Medium (DMEM) containing 10% (v/v) fetal bovine serum, 2 mM glutamine, 5 U/ml penicillin, and 50 μg/ml streptomycin at 37°C in a humidified O_2_/CO_2_ (19:1) atmosphere. NCTC cells were cultured in Dulbecco’s Modified Eagle Medium (DMEM) containing 10% (v/v) fetal horse serum, other conditions are the same as HepG2.

### Animal model and experimental protocol

Male C57BL/6 mice weighing 25 ± 0.5g (mean ± SD) were bought from the animal quarters at the Zhejiang Traditional Chinese Medical University and housed in there. The mice were divided into three groups (n = 8 per group): pair-fed (PF) group, alcohol-fed (AF) group and alcohol-fed/Danshen (AFD) group. PF group were maintained on an isocaloric control liquid diet (Bioserv, Frenchtown, NJ) for 5 weeks. AF group were fed ad libitum with an ethanol-containing Lieber-DeCarli diet (ethanol-derived calories were increased from 30% to 36% during the first 4 weeks, with a 2% increase each week) for 5 weeks. In comparison, mice in AFD group were fed the same ethanol-containing Lieber-DeCarli diet as above, and were given an intraperitoneal injection of Danshen at a dose of 3g/kg body weight every day from week 2 until the end of the diet program. Food intake and body weight were recorded daily and weekly, respectively. At the end of the experiment, the mice were anesthetized with Avertin (300 mg/kg body weight) after 4 hours of fasting, and all efforts were made to minimize suffering. Plasma, liver, and epididymal fat pad samples were harvested for assays. The local ethics committees for animal experimentations approved all experiments.

### Lactate dehydrogenase (LDH) assay

Cell death was determined by the release of LDH into the culture medium. LDH activity was determined spectrophotometrically at 340 nm using a commercially available kit. LDH assay kit was purchased from Thermo scientific (Middletown, VA).

### Liver triglyceride measurement

Liver tissue were homogenized and hepatic total lipids were extracted. Hepatic triglyceride content was determined via enzymatic colorimetric methods using commercially available kits. Triglycerides assay kit was purchased from Nanjing Jiancheng Bioengineering Institute (Nanjing, China).

### Histological examination

H&E staining of liver sections was performed as previously described [32]. At the time of killing, the liver was harvested and small pieces were fixed immediately in 10% buffered formalin. After paraffin embedding, 5μm sections were deparaffinized in xylene and were rehydrated through series of decreasing concentrations of ethanol. Sections were stained with hematoxylin-eosin. In short, formalin-fixed, paraffin-embedded liver tissue sections were deparaffinized and then rehydrated in 100%, 95%, and 70% graded ethanol washes.

### Quantitative real-time reverse transcription (RT)-PCR

Total RNA, from either frozen liver tissue or cultured cells, was isolated with a phenol-chloroform extraction. For each sample, 1μg total RNA was reverse transcribed using a high-capacity cDNA reverse transcription kit (Applied Biosystems, Foster City, CA). The cDNA was amplified in MicroAmp Optical 96-well reaction plates with a SYBR Green PCR Master Mix (Applied Biosystems) on an Applied Biosystems Prism 7000 sequence detection system. Relative gene expression was calculated after normalization by a house-keeping gene (mouse or human 18S rRNA). The relevant primer sequences are shown in Table 1.

**Table 1:** Primer sequences.

Name	Sequence
GSTA4	Sense: 5’-AACTTGTATGGGAAGGACCTGAA-3’
	Anti-sense: 5’-CCACGGCAATCATCATCATC-3’
CPT1	Sense:5’-TCCAGTTGGCTTATCGTGGTG-3’
	Anti-sense:5’-CTAACGAGGGGTCGATCTTGG-3’
CPT2	Sense:5’-ACCTGGTCAATGCGTATCCC-3’
	Anti-sense:5’-ACTGCCGAGTCCACTTTCCT-3’
18S	Sense:5’-ATACATGCCGACGGGCGCTG-3’
	Anti-sense:5’-CGGCTCGGGCCTGCTTTGAA-3’

### Western blotting analysis

Tissues or cells were lysed in RIPA buffer and the isolated proteins were separated by SDS polyacrylamide gel electrophoresis and transferred to 0.45μm polyvinylidene difluoride (PVDF) membranes. After transferring, membranes were blocked in 1% BSA in PBS with 0.1% Tween-20 and probed with primary antibodies, and then with a horseradish peroxidase-conjugated secondary antibody. Target protein detection was performed using the Enhanced Chemiluminescence Substrate Kit (BROSTER, Wuhan, China).

### Statistical analysis

All data were expressed as mean ± SD. Statistical analysis was performed using a one-way analysis of variance (ANOVA) and further analyzed by Newman—Keuls test for statistical difference. Differences between treatments were considered to be statistically significant at *p* < 0.05.

## Results

### Danshen administration improves liver pathological changes of early-stage ALD in mice

**Fig 1:**
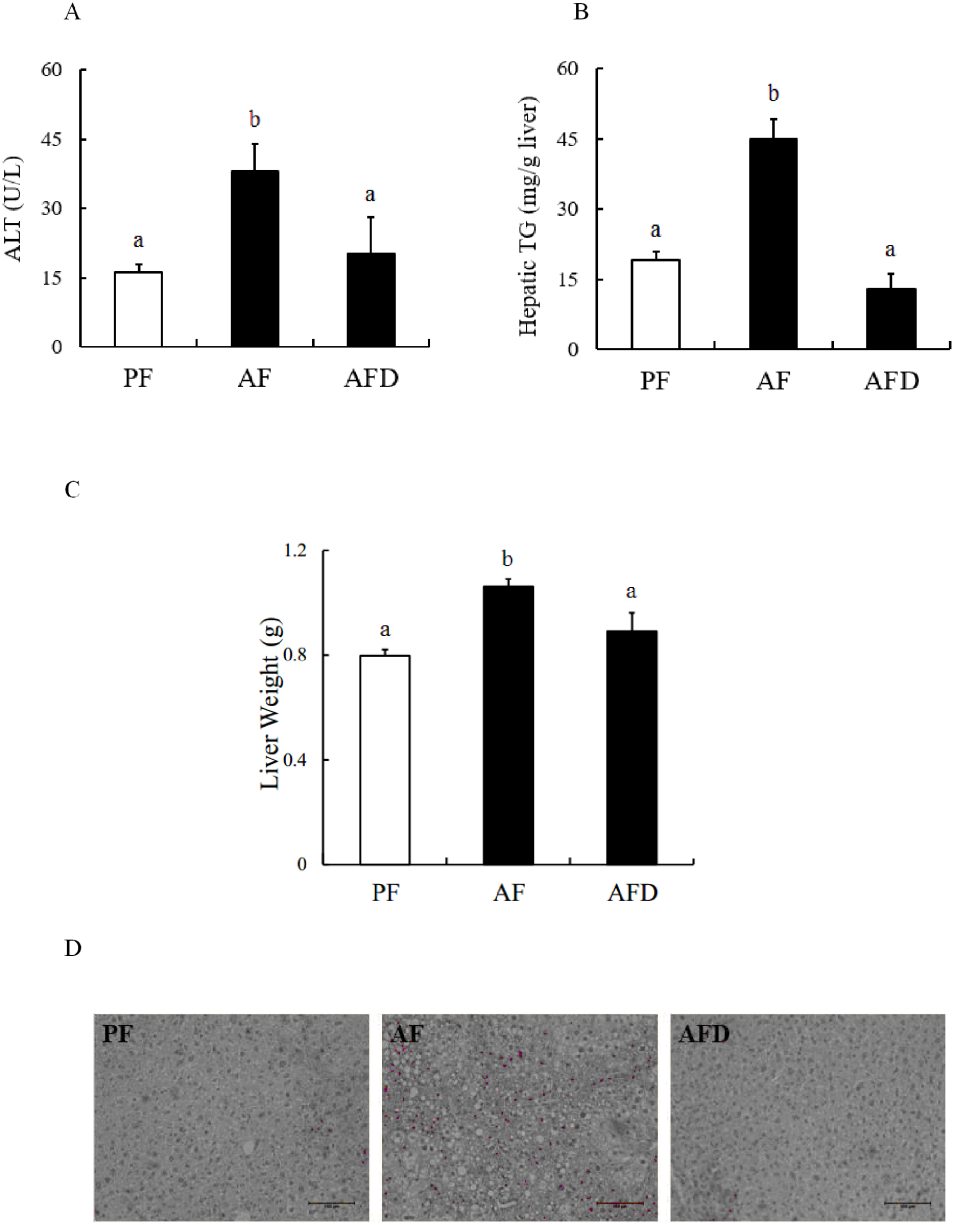
Danshen alleviates liver injury and intrahepatic TG accumulation resulted from chronic alcohol exposure. **A**. Serum alanine aminotransferase (ALT) levels. **B**. Hepatic triglyceride (TG) contents. **C**. Liver weights. **D**. H&E staining of liver tissues. Data are expressed as the mean ± SD (n = 8 mice per group). Bars with different letters differ significantly (p < 0.05). PF: pair feeding; AF: alcohol feeding; AFD: alcohol feeding with Danshen injection.

The early-stage ALD, characterized by fatty liver and mild-grade liver damage, was established by feeding male C57BL/6 mice (8-week old) with the Lieber-DeCarli alcohol-containing liquid diet for 5 weeks. The effects of Danshen on alcohol-induced liver pathologies were investigated by i.p. injection of Danshen (3g/kg BW, daily) as described in Materials and Methods, which was initiated at week 2. In line with many previous studies, including ours, 5-week alcohol-containing diet feeding resulted in liver injury and fatty liver in mice, evidenced by significantly increased plasma ALT levels (Fig 1A), hepatic triglyceride contents (Fig 1B), and the weight of livers (Fig 1C). Moreover, H&E staining illustrated a massive hepatic fat accumulation in alcohol-fed mice. When Danshen was administered to alcohol-fed mice, both hepatic TG contents, liver weights and plasma ALT levels were significantly decreased (Fig 1A–1D), suggesting Danshen administration confers protection against early-stage ALD.

### Danshen administration suppresses alcohol-induced hepatic 4-HNE accumulation

**Fig 2:**
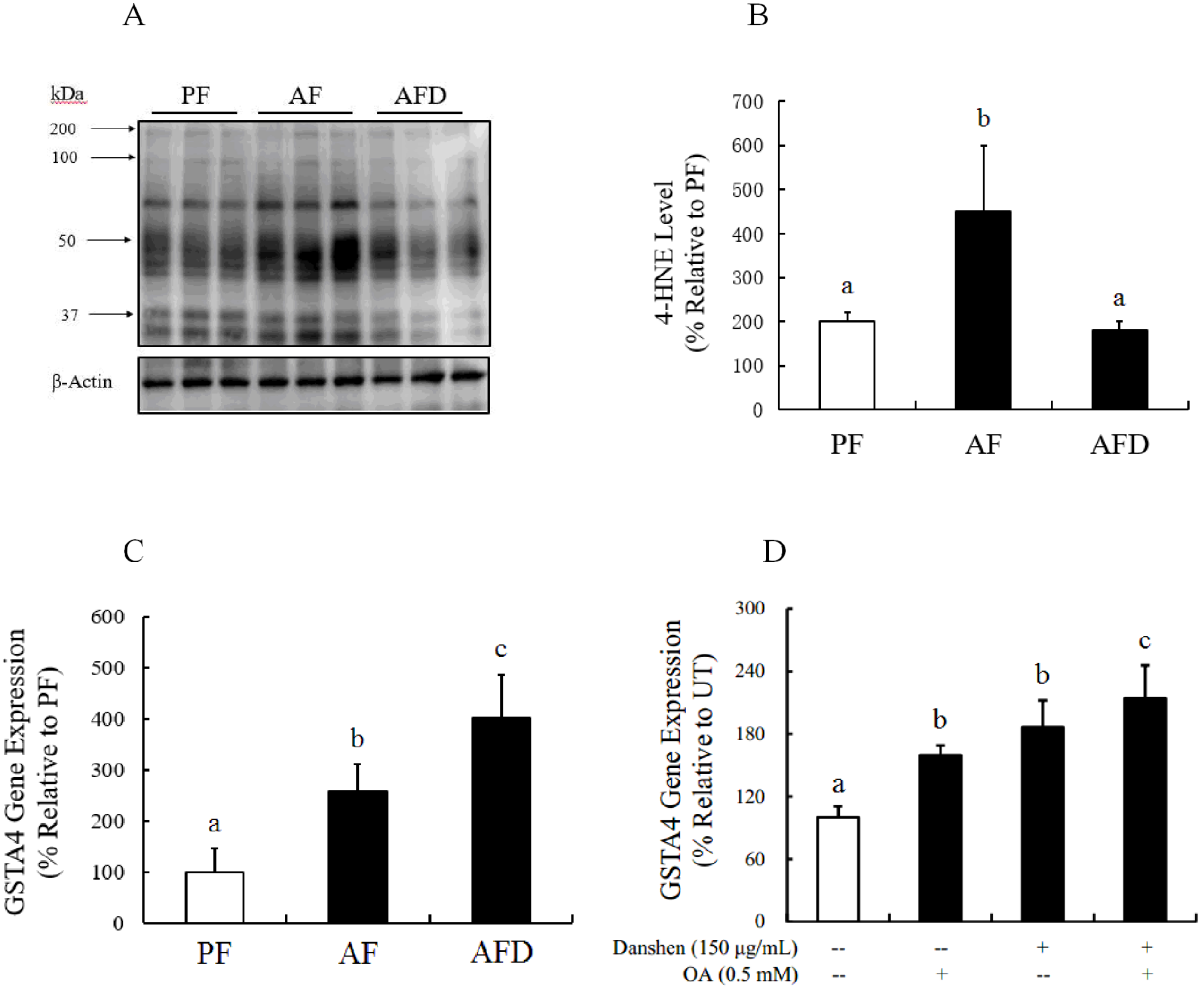
Danshen inhibits 4-HNE expression in mice fed an ethanol-containing Lieber-DeCarli diet. **A**. Protein expression of 4-HNE in various groups of mice as indicated by Western Blotting. **B**. The expression of 4-HNE. **C**. Gene expression level of GSTA4 in different groups of mice. Data are expressed as the mean ± SD (n = 8 mice per group). Bars with different letters differ significantly (p < 0.05). PF: pair feeding; AF: alcohol feeding; AFD: alcohol feeding with Danshen injection. **D**. Gene expression level of GSTA4 in NCTC1469 cells treated with Danshen at 150 μg/mL and/or Oleate (OA) at 0.5 mM. All values are denoted as the mean ± SD from three or more independent studies. Bars with different letters differ significantly (p < 0.05).

We previously reported that 4-HNE accumulation in hepatocytes contributes to alcohol-induced hepatocyte cell death via suppressing hepatocyte NF-κB activation [22]. The effects of Danshen administration on hepatic 4-HNE contents as well as NF-κB activation in the setting of chronic alcohol exposure were subsequently examined. As shown in Fig 2A & 2B, chronic alcohol exposure was associated with increased hepatic 4-HNE accumulation, which was concomitant with suppressed NF-κB activation, as evidenced by decreased nuclear NF-κB p65 abundance. This observation was consistent with our previous report [22]. Importantly, we found in this study that Danshen administration ameliorated alcohol-triggered 4-HNE accumulation in the liver (Fig 2A & 2B) and prevented alcohol-induced hepatic NF-κB suppression (Fig 2C & 2D).

### Danshen prevents alcohol-induced hepatic PPARα suppression

**Fig 3:**
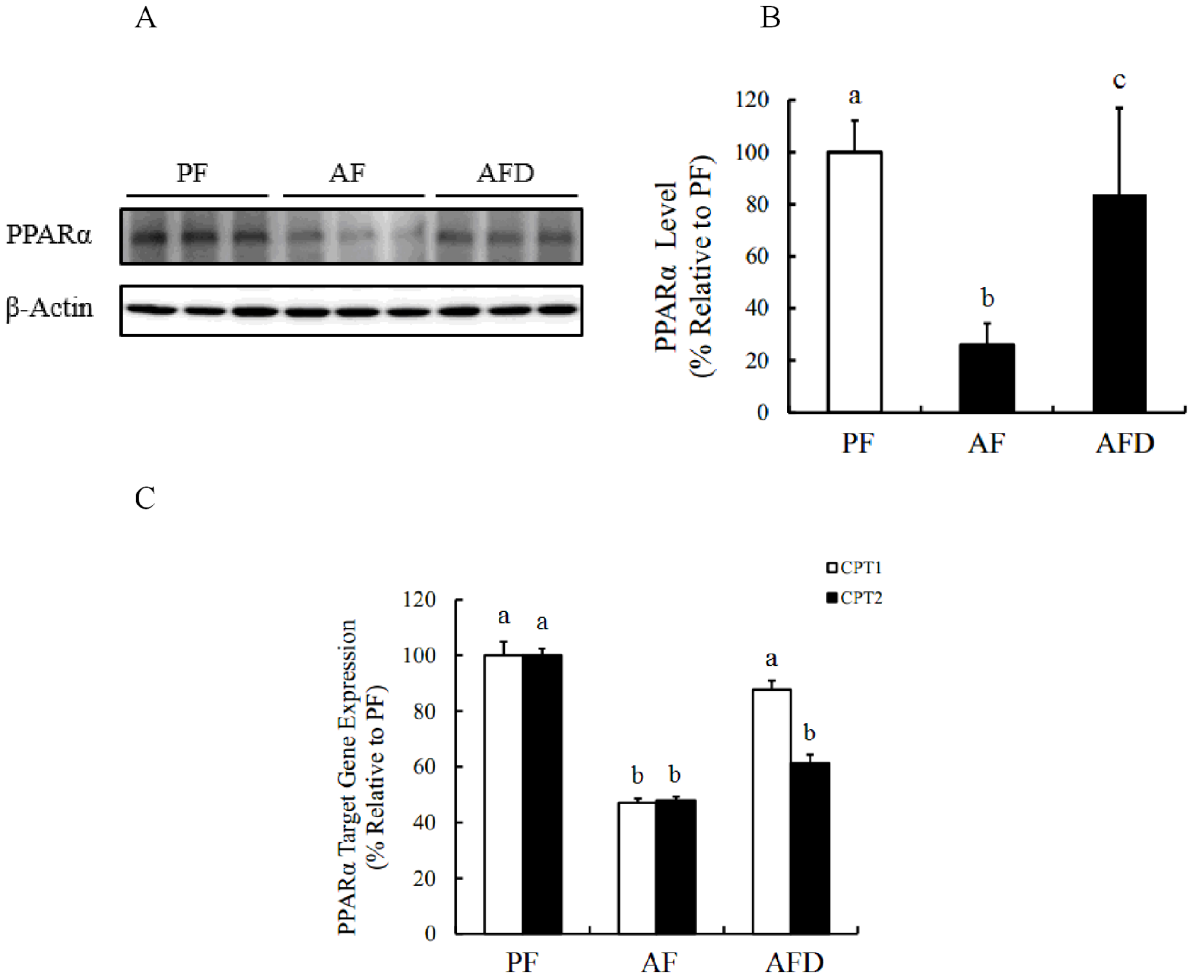
Danshen prevents long-term alcohol exposure induced deactivation of PPARα in mice. **A**. PPARα protein expression levels in liver tissue extracts of mice were measured by Western Blotting. **B**. The level of PPARα. **C**. Gene expression levels of CPT-1 and CPT-2 in different groups of mice. Data are expressed as the mean ± SD (n = 8 mice per group). Bars with different letters differ significantly (p < 0.05). PF: pair feeding; AF: alcohol feeding; AFD: alcohol feeding with Danshen injection.

Peroxisome proliferator-activated receptor alpha (PPARα) is a nuclear hormone receptor protein with central roles in the modulation of lipid synthesis, transport and fatty acid metabolism [5–8]. To test if the observed beneficial effects of Danshen supplementation in our study are related to its alleviative effects on alcohol-induced hepatic PPARα suppression, we measured the regulatory effect of Danshen supplementation on PPARα in liver tissue by Western blotting and (RT)-PCR. As show in Fig 3, chronic alcohol exposure suppressed PPARα protein productions, and Danshen administration increased alcohol-induced PPARα suppression in the liver (Fig 3A & 3B). We next measured the expression levels of two downstream target genes of PPARα, carnitine palmitoyl transferase 1 and 2 (CPT-1 and CPT-2). The mRNA expression levels of CPT-1 and 2 were significantly suppressed by chronic alcohol exposure, however, the addition of Danshen was shown to be able to partially increased the expression of CPT-1 and 2 (Fig 3C), suggesting that the activation of PPARα may play an important role in Danshen’s beneficial effects on alcohol-induced cell death.

### Danshen supplementation protects hepatocytes from 4-HNE-elicited cell death

**Fig 4:**
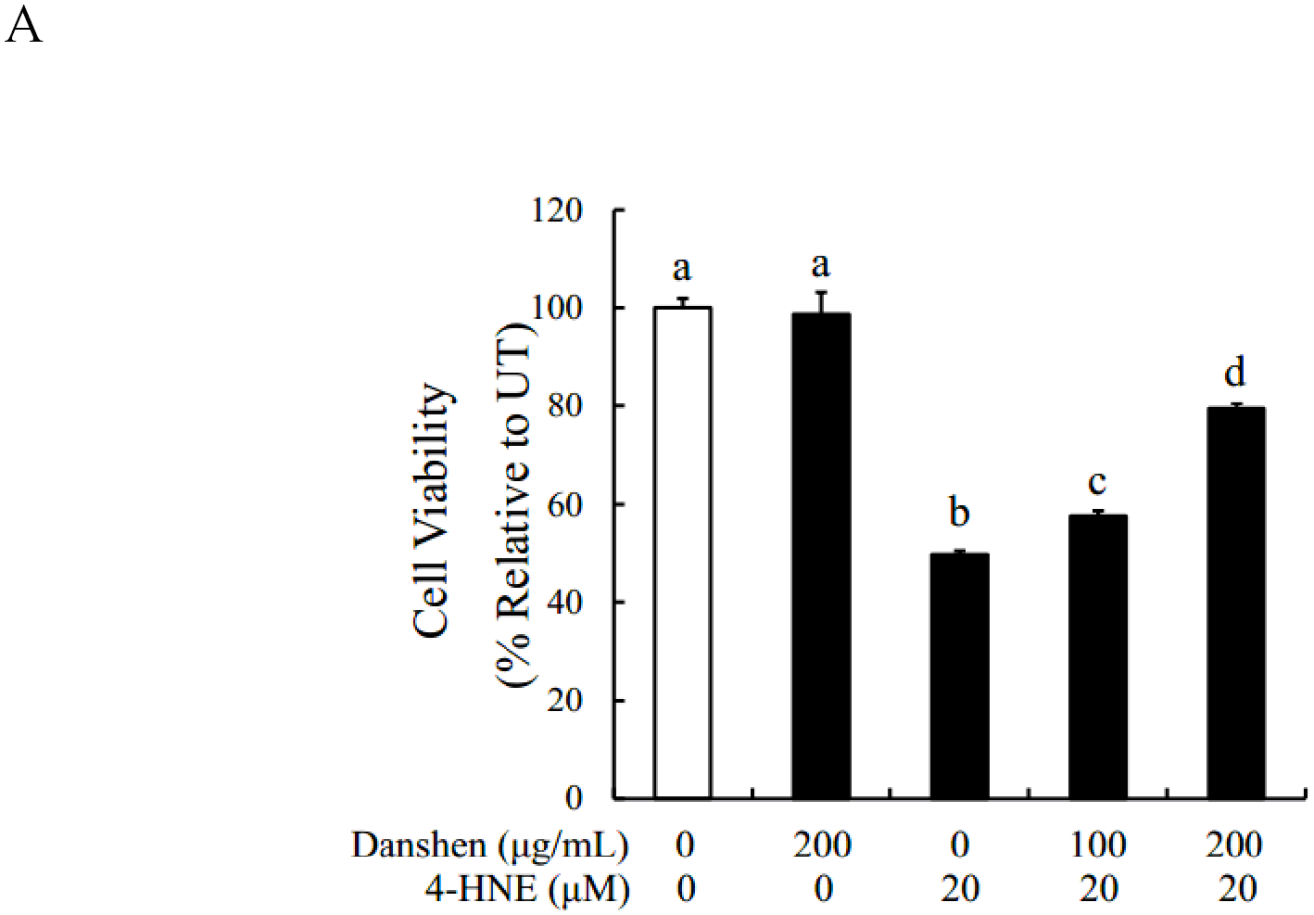
Danshen supplementation protects hepatocytes from 4-HNE-elicited cell death. **A**. Danshen significantly prevents 4-HNE induced LDH release from HepG2 cells. HepG2 cells are pretreated with 100 or 200 μg/mL Danshen for 2 hours before 4-HNE addition. LDH release is measured 16 hours later. All values are denoted as the mean ± SD from three or more independent studies. Bars with different letters differ significantly (p < 0.05).

To determine the effect of Danshen on 4-HNE induced cell death, the cell activity of Danshen supplementation on 4-HNE treated hepatocellular was measured. As show in Fig 4, Danshen suppressed 4-HNE-induced cell death, evidenced by a significant reduction of LDH release in comparison to 4-HNE alone treatment (Fig 4A).

### PPARα activation contributes the hepatoprotective effect of Danshen against 4-HNE hepatotoxicity

**Fig 5:**
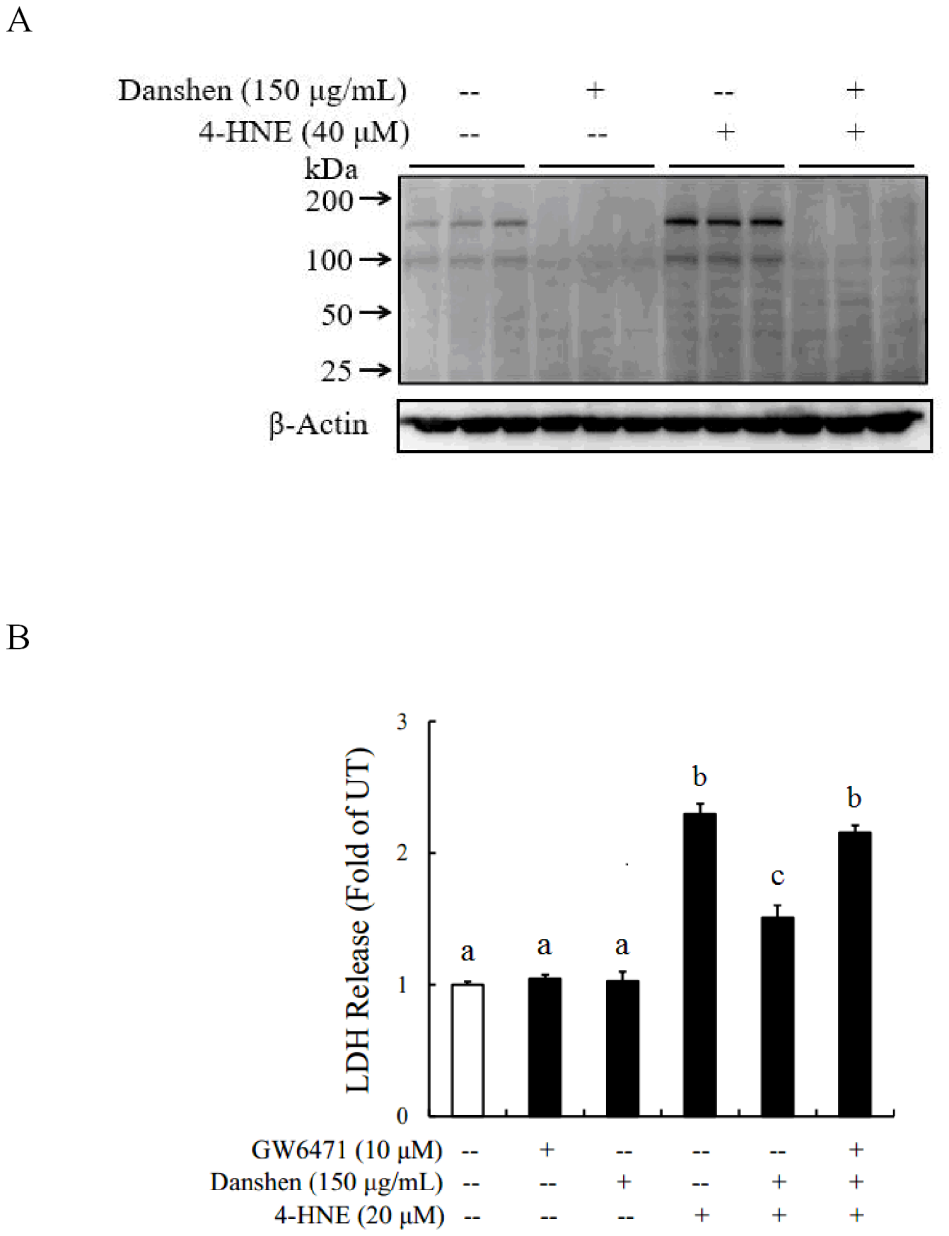
PPARα activation contributes the hepatoprotective effect of Danshen against 4-HNE hepatotoxicity. **A**. Danshen significantly reduces the intracellular 4-HNE accumulation after exogenous 4-HNE exposure. NCTC1469 cells are pretreated with 150μg/mL Danshen for 2 hours before 4-HNE addition. Cell lysates were collected 8 hours later for Western Blotting analysis. **B**. GW6471, PPARα antagonist, significantly increased cell death induced by 4-HNE in HepG2 cells. HepG2 cells are pretreated with GW6471 for 1 hour, then Danshen was treated 2 hours before 4-HNE exposure. LDH release is measured 16 hours later. All values are denoted as the mean ± SD from three or more independent studies. Bars with different letters differ significantly (p < 0.05).

To determine whether the hepatoprotective effect of Danshen against 4-HNE hepatotoxicity was mediated by the activation of PPARα, we measured intracellular 4-HNE levels after Danshen treatment, to determine whether Danshen affected intracellular 4-HNE metabolism. As show in Fig 5, the intracellular 4-HNE accumulation was increased after 4-HNE exposure, and Danshen treatment significantly reduced this accumulation (Fig 5A). To further verify the association between PPARα and 4-HNE formation, GW6471, PPARα antagonist that inhibits activation of PPARα was also used. As shown in Fig 5B, Danshen decreased the cell death that induced by 4-HNE, however, GW6471 reversed this process (Fig 5B).

### Danshen promotes cellular 4-HNE catabolism via activating PPARα

**Fig 6:**
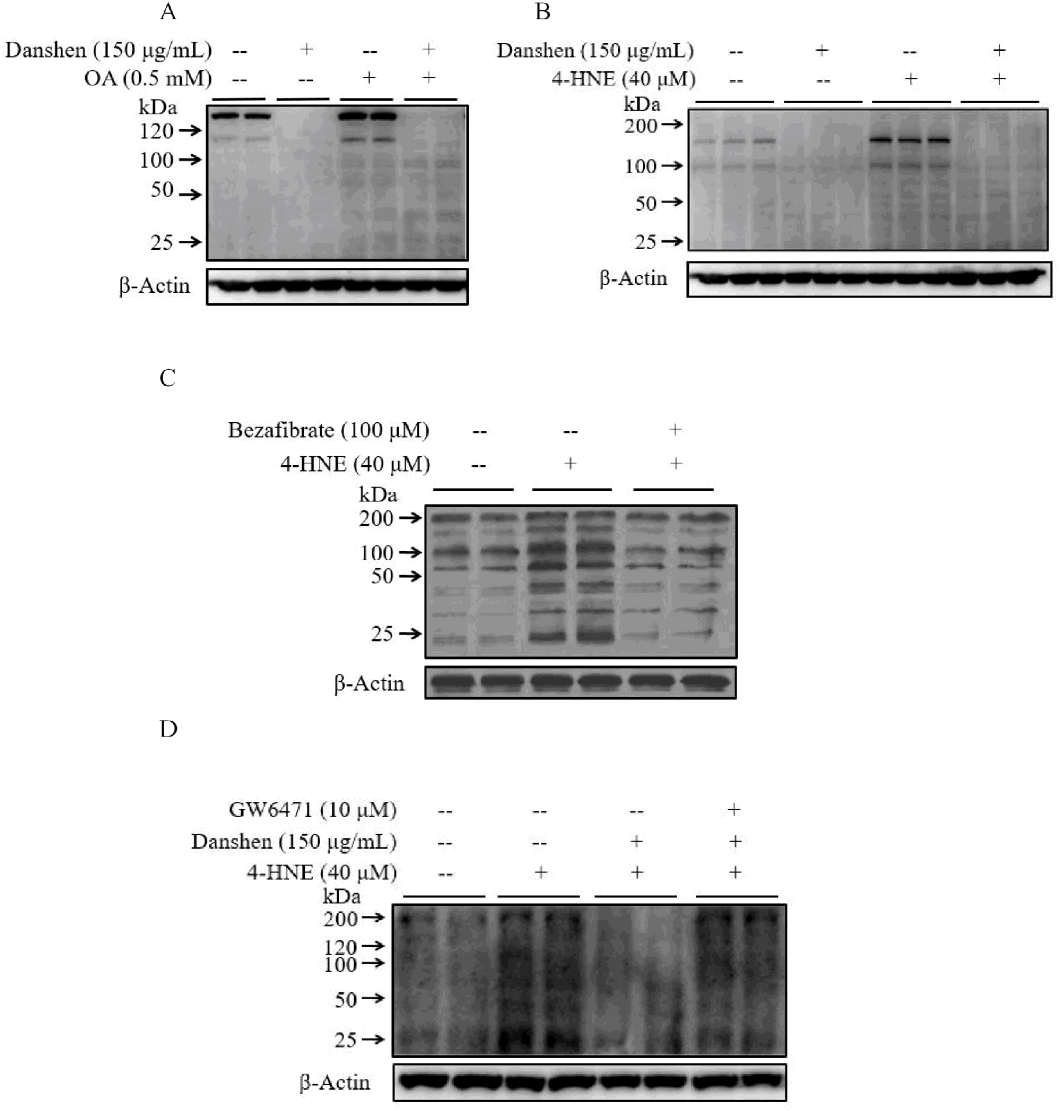
Danshen promotes the catabolism of 4-HNE via activating PPARα. **A & B**. Danshen significantly reduces the intracellular 4-HNE accumulation after exogenous 4-HNE or OA exposure. NCTC1469 cells are pretreated with 150μg/mL Danshen for 2 hours before 4-HNE or OA addition. Cell lysates were collected 8 hours later for Western Blotting analysis. **C**. Bezafibrate, PPARα agonists, significantly decreased the intracellular 4-HNE accumulation after exogenous 4-HNE exposure. HepG2 cells are pretreated with 100 μM bezafibrate for 1 hour before 4-HNE exposure. Cell lysates were collected 8 hours later for Western Blotting analysis. **D**. GW6471, PPARα antagonist, significantly increased the intracellular 4-HNE accumulation after exogenous 4-HNE exposure which has been decreased after Danshen addition. HepG2 cells are pretreated with GW6471 for 1 hour, then Danshen was treated 2 hours before 4-HNE exposure. Cell lysates were collected 8 hours later for Western Blotting analysis.

To understand the possible mechanisms by which Danshen prevents ethanol-induced liver injury, the effect of Danshen supplementation on ethanol-induced 4-HNE accumulation in the hepatocellular was determined. As shown in Fig 6, the intracellular 4-HNE accumulation was increased after 4-HNE or oleate (OA) exposure, both Danshen and bezafibrate significantly reduced this accumulation (Fig 6A–6C). On the contrary, GW6471 prevent the protective effect of Danshen on the exposure of 4-HNE in hepatocytes (Fig 6D), suggesting that Danshen significantly diminished ethanol-induced 4-HNE accumulation via activating of PPARα.

## Discussion

The goal of this study was to determine the potential beneficial effect of Danshen administration on ALD and to clarify the underlying molecular mechanisms. Using the well-established Lieber-DeCarli mouse model of ALD, we demonstrate that Danshen administration improves liver pathologies of ALD, including fat accumulation and liver damage. Specifically, our data show that Danshen administration attenuates alcohol-induced increment of hepatic 4-HNE formation and prevented hepatic PPARα suppression in response to chronic alcohol consumption. In cultured hepatocytes, Danshen supplementation prevented 4-HNE-induced cell death, which is concomitant with accelerated intracellular 4-HNE degradation. Further mechanistic investigations reveal that both hepatoprotective effect and increased intracellular 4-HNE clearance instigated by Danshen supplementation are PPARα-dependent, as both are abolished by the pretreatment of hepatocytes with GW6471, a PPARα inhibitor. Meanwhile, bezafibrate, a potent PPARα agonist, confers protection against 4-HNE-induced cell death in hepatocytes. Taken together, our data suggest that Danshen administration can protect against ALD via inducing PPARα activation and subsequent 4-HNE degradation.

Oxidative stress plays a central and casual role in the onset and progression of the disease development. Chronic ethanol exposure increases the production of reactive oxygen species (ROS), and lowers cellular antioxidant levels, leading to oxidative stress. Lipid peroxidation, oxidative decomposition of polyunsaturated fatty acids (PUFAs) elicited by ROS, is one of the direct consequences and the hallmark of intracellular oxidative stress. The aldehydic molecules formed during lipid peroxidation are the ultimate mediator of toxic effects elicited by oxidative stress. Of lipid peroxides, 4-HNE is among the most abundant and reactive aldehydic products derived from peroxidation of n-6 PUFAs. Convincing evidence, including these from ours, supports that hepatic 4-HNE accumulation contributes to the pathogenesis of ALD. An elevation of intracellular 4-HNE accumulation may result from an imbalance between 4-HNE formation and its degradation. In the present study, we examined the effect of Danshen administration on hepatic 4-HNE formation in the setting of chronic alcohol exposure. We showed that Danshen administration in mice prevented alcohol-induced hepatic 4-HNE accumulation. Considering previously reported antioxidant property of Danshen, it is rationale to posit that Danshen administration is capable of alleviating cellular 4-HNE accumulation via acting as an antioxidant to prevent 4-HNE formation. In the present study, we also explored the potential effect of Danshen supplementation on 4-HNE degradation via directly adding exogenous 4-HEN into the cell culture medium. We showed clearly that the pretreatment of hepatocytes with Danshen ameliorated intracellular 4-HNE elevation in response to exogenous 4-HNE addition, suggesting that Danshen can reduce intracellular accumulation of 4-HNE by increasing it degradation.

PPARα is a nuclear factor and expressed predominantly in tissues that have a high level of fatty acid catabolism, and regulates diverse aspects of lipid metabolism, including the induction of cellular fatty acid uptake and β-oxidation of fatty acids, thereby modulating hepatic fatty acid synthesis and plasma triglyceride and cholesterol concentrations [33]. Decreased hepatic PPARα expression and transactivity by chronic alcohol exposure has been reported and PPARα activation significantly reduced liver injury in mice that induced by long-term alcohol exposure [34]. We previously reported that other than regulating lipid metabolism, PPARα activation was also critically involved in the regulation of 4-HNE catabolism [22]. In consistent with our previous report, we observed in the present study that chronic alcohol consumption was associated with hepatic PPARα suppression and its activation by bezafibrate in hepatocytes attenuated intracellular 4-HNE accumulation in response to exogenous 4-HNE supplementation. Interestingly, we found that Danshen administration prevented alcohol-induced PPARα suppression in mice. Moreover, both cytoprotective and 4-HNE-lowering effects of Danshen supplementation were abolished by PPARα inhibitor in hepatocytes exposed to exogenous 4-HNE, suggesting that PPARα activation plays a mechanistic role in Danshen’s action to promote 4-HNE degradation and confer cellular protection.

The degradation of intracellular lipid peroxides involves multiple enzyme systems and each of which has a variety of isoforms. It is possible that PPARα activation may collectively upregulate several enzymes involved in 4-HNE metabolism. Among these is glutathione S-transferase A4 (GSTA4), which promotes 4-HNE degradation via in conjugation with Glutathione (GSH) and a target protein of PPARα activation in the liver [35–39]. In this study, the observations that Danshen upregulated GSTA4 expression in both the livers of alcohol-fed mice and cultured hepatocytes with/without oleic acid exposure suggests this enzyme may be a downstream target of Danshen administration, attributing to its 4-HNE-reducing action. As previous report demonstrated that GSTA4/PPARα double knockout mice manifested more pronounced hepatic 4-HNE protein adducts than single knockout ones during early stages of alcoholic liver disease [40], it is very likely that PPARα activation-promoted 4-HNE degradation involves upregulation/mobilization of multiple enzyme systems, which warrant further investigation. In conclusion, our study demonstrates that Danshen administration can prevent ALD in mice potentially via PPARα activation-dependent hepatic 4-HNE clearance. These beneficial changes were attributable to Danshen can reduce intracellular accumulation of 4-HNE by increasing it degradation. Furthermore, the activation of PPARα may collectively upregulate several enzymes involved in 4-HNE metabolism, suggesting that PPARα activation plays a mechanistic role in Danshen’s action to promote 4-HNE degradation and confer cellular protection. Therefore, Danshen could serve as an ideal therapeutic agent for ALD. Our study provides evidence for further evaluation of the potential therapeutic role of Danshen in ALD.

## Supporting information

S1 Dataset Relevant data underlying the findings described in manuscript. (ZIP)

## Abbreviations

4-HNE: 4-hydroxynonenal
ALD: alcoholic liver disease
CPT-1: carnitine palmitoyl transferase 1
CPT-2: carnitine palmitoyl transferase 2
GSH: Glutathione
GSTA4: glutathione S-transferase A4
LDH: lactate dehydrogenase
NF-κB: nuclear factor-kappa-binding
PPARα: peroxisome proliferators activated receptor alpha
PUFAs: polyunsaturated fatty acids
ROS: reactive oxygen species
TNF-α: tumor necrosis factor-alpha
