# Supplementary material for: Danshen protects against early-stage alcoholic liver disease in mice via inducing PPARα activation and subsequent 4-HNE degradation
Source: PLoS One. 2017 Oct 11;12(10):e0186357. doi: 10.1371/journal.pone.0186357 (PMC5636149; doi:10.1371/journal.pone.0186357)
Supplement: S1 Dataset — (ZIP) [file pone.0186357.s001.zip › S1 Dataset/Fig S2A-B.pptx]

## Slide 1
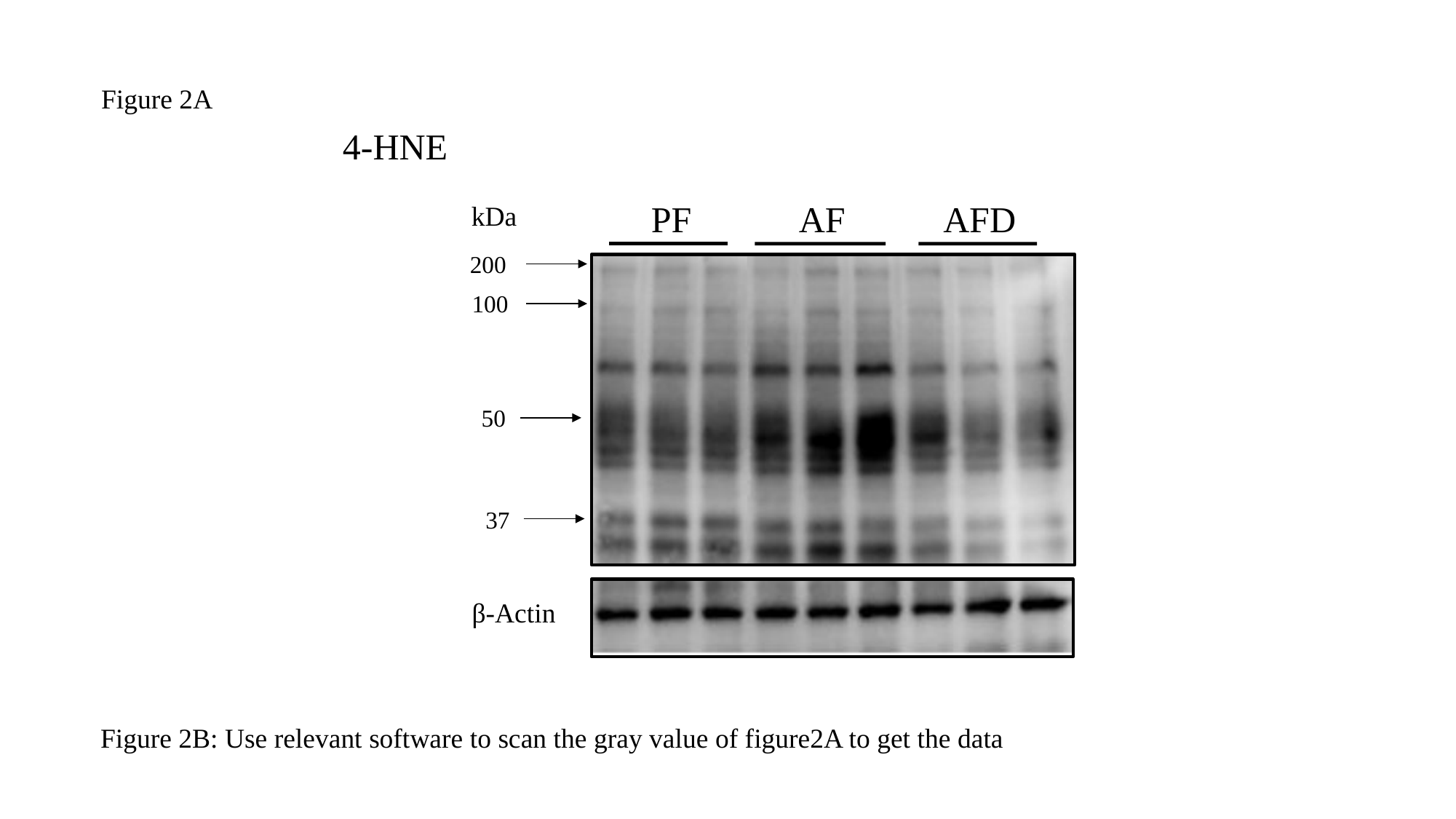

Figure 2A
4-HNE
 PF AF AFD
kDa
200
100
50
37
β-Actin
Figure 2B: Use relevant software to scan the gray value of figure2A to get the data
