# Supplementary material for: Danshen protects against early-stage alcoholic liver disease in mice via inducing PPARα activation and subsequent 4-HNE degradation
Source: PLoS One. 2017 Oct 11;12(10):e0186357. doi: 10.1371/journal.pone.0186357 (PMC5636149; doi:10.1371/journal.pone.0186357)
Supplement: S1 Dataset — (ZIP) [file pone.0186357.s001.zip › S1 Dataset/Fig S6.pptx]

## Slide 1
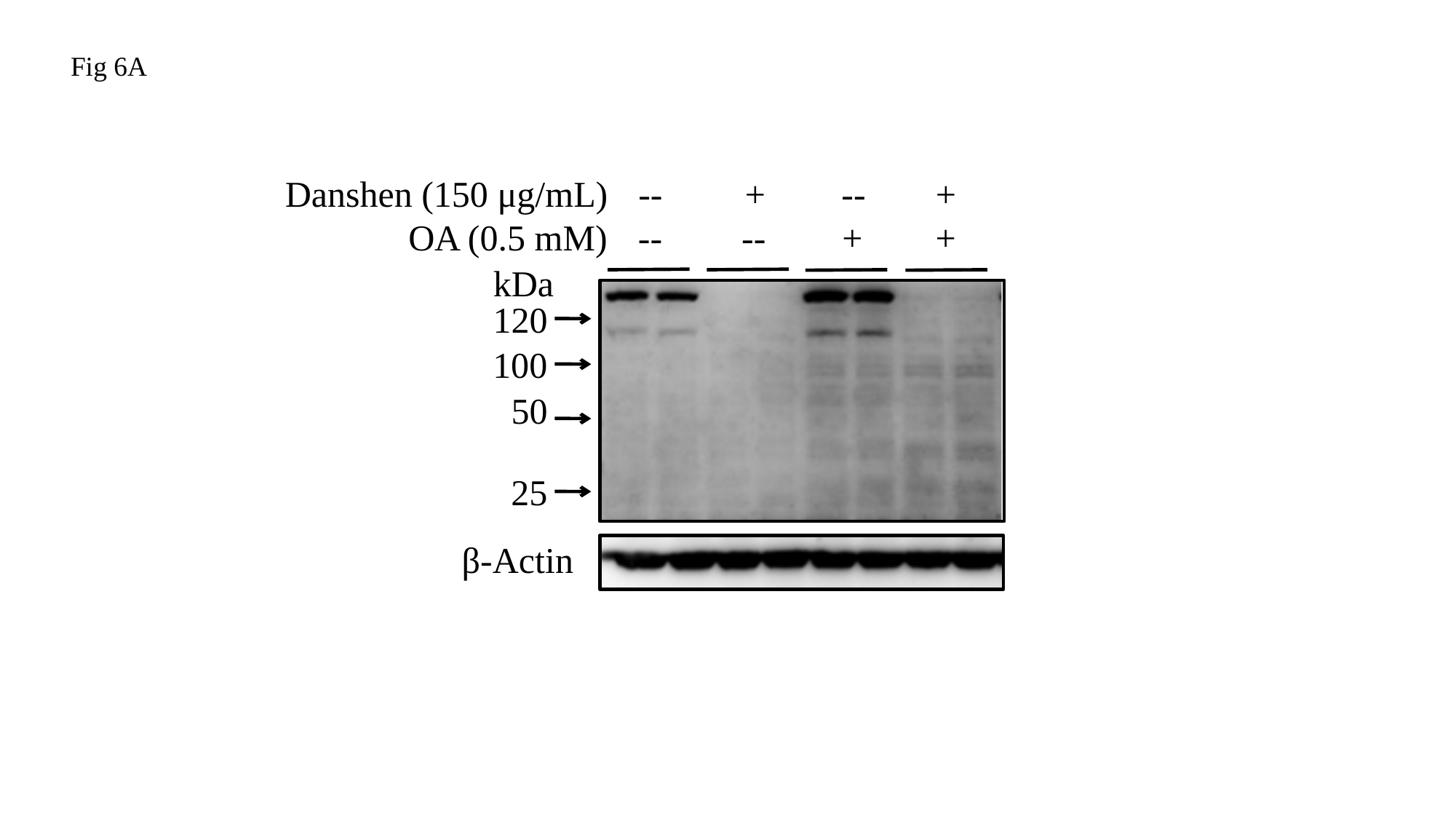

Fig 6A
Danshen (150 μg/mL) -- + -- +
 OA (0.5 mM) -- -- + +
kDa
120
100
50
25
β-Actin

## Slide 2
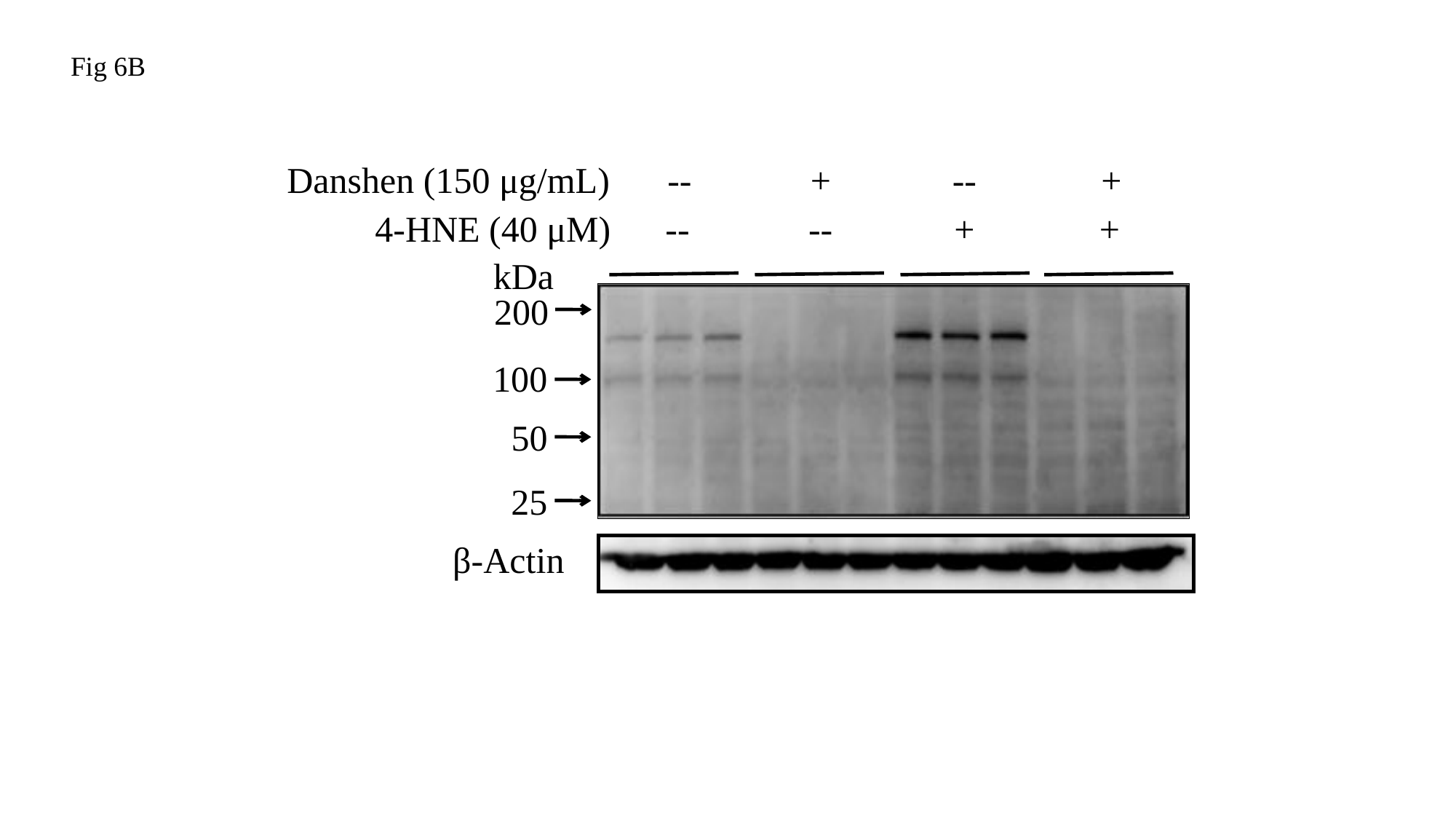

Fig 6B
Danshen (150 μg/mL) -- + -- +
 4-HNE (40 μM) -- -- + +
kDa
200
100
50
25
β-Actin

## Slide 3
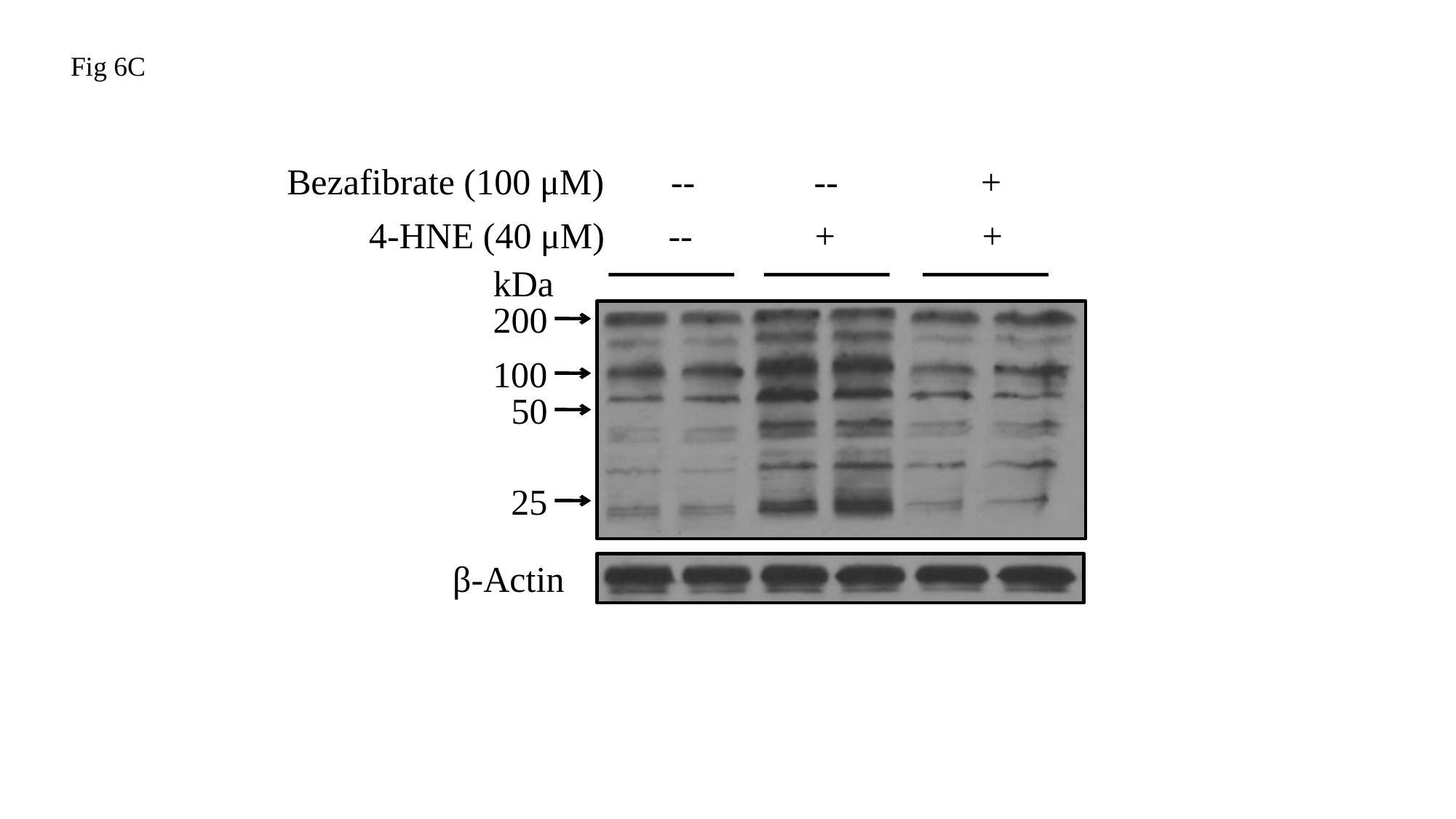

Fig 6C
Bezafibrate (100 μM) -- -- +
 4-HNE (40 μM) -- + +
kDa
200
100
50
25
β-Actin

## Slide 4
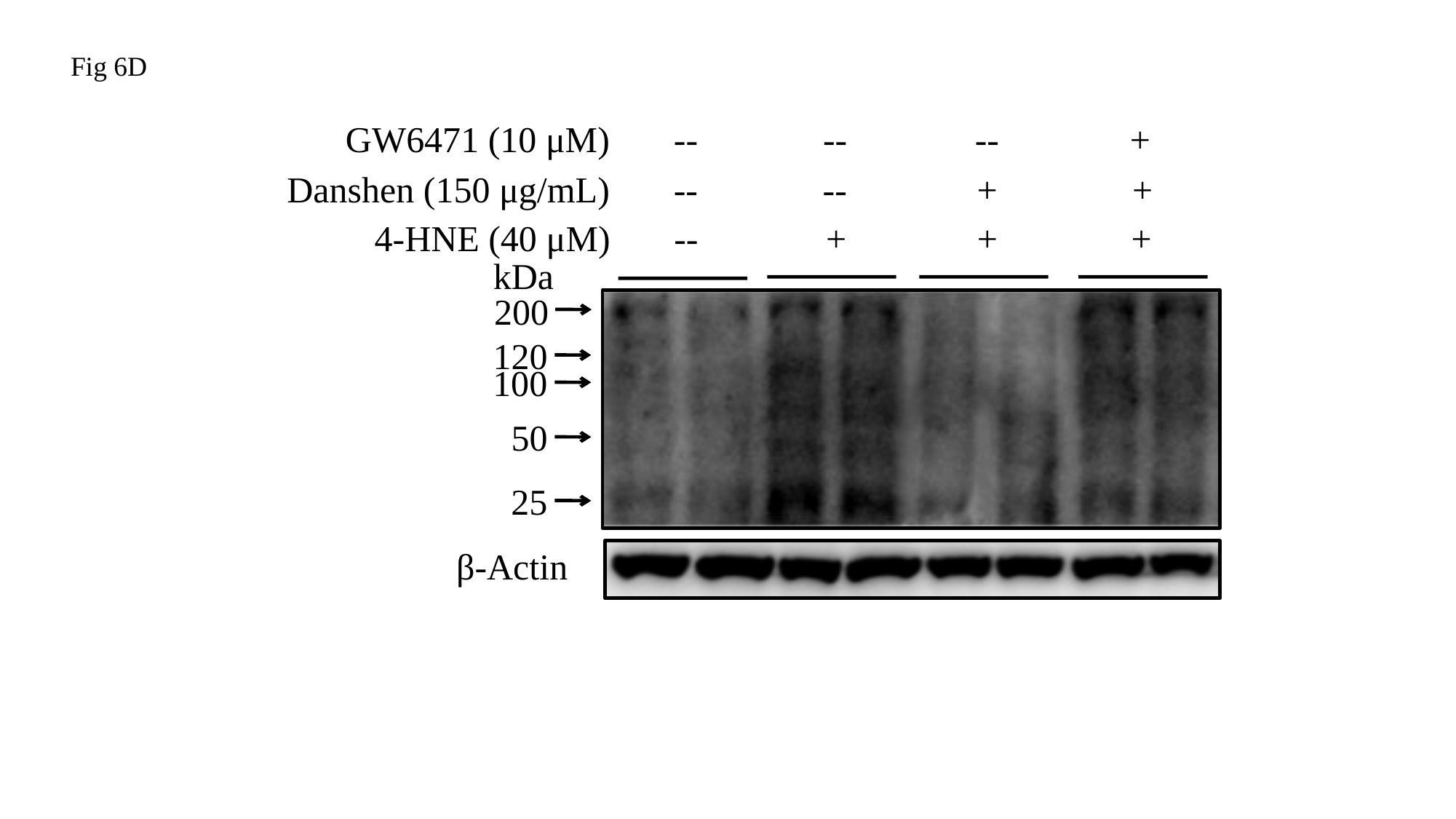

Fig 6D
GW6471 (10 μM) -- -- -- +
Danshen (150 μg/mL) -- -- + +
 4-HNE (40 μM) -- + + +
kDa
200
120
100
50
25
β-Actin
