# Supplementary figures and images for: Danshen protects against early-stage alcoholic liver disease in mice via inducing PPARα activation and subsequent 4-HNE degradation
Source: PLoS One. 2017 Oct 11;12(10):e0186357. doi: 10.1371/journal.pone.0186357 (PMC5636149; doi:10.1371/journal.pone.0186357)

## Slide 1
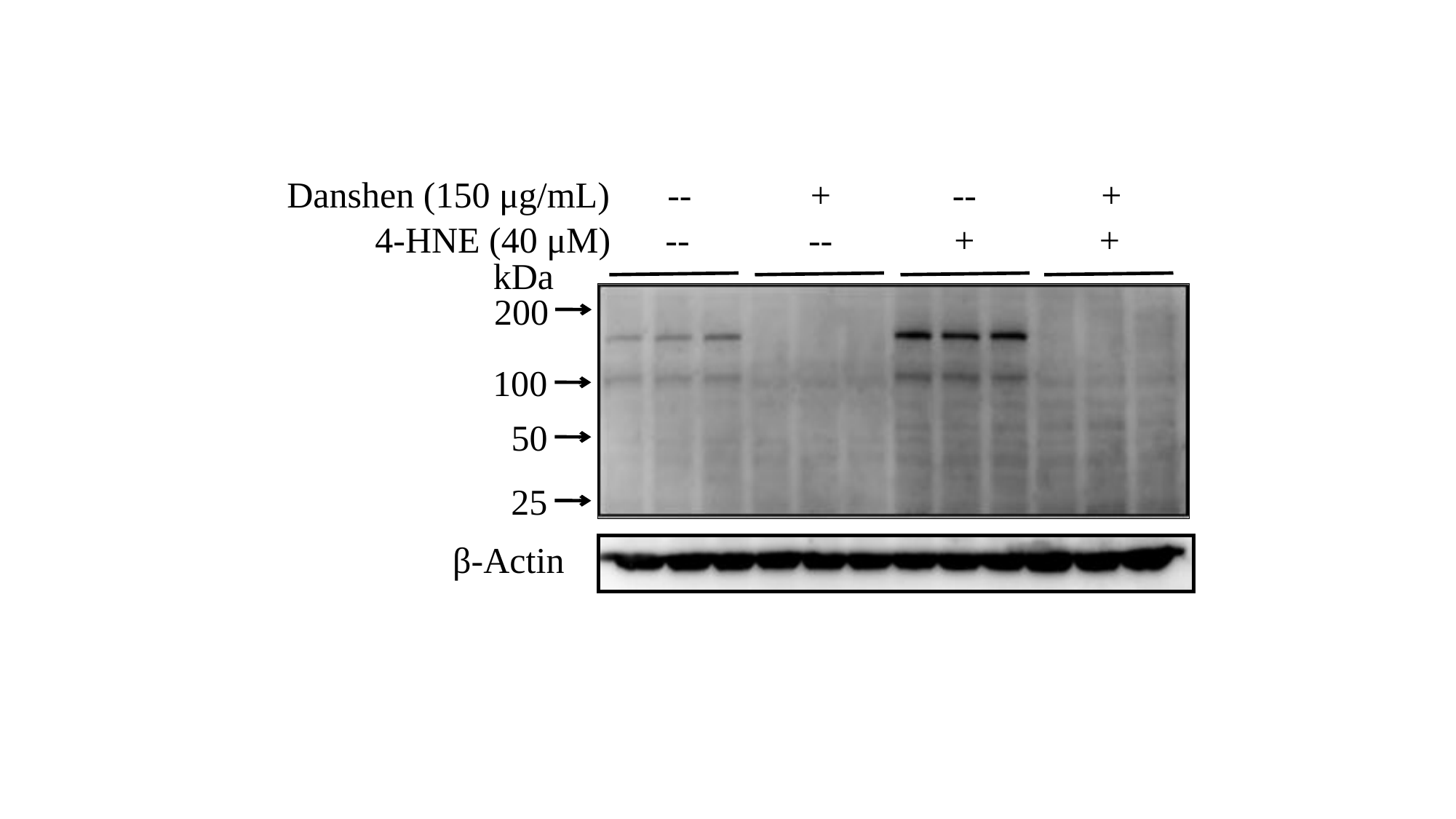

Danshen (150 μg/mL) -- + -- +
 4-HNE (40 μM) -- -- + +
kDa
200
100
50
25
β-Actin

Supplement: S1 Dataset — (ZIP) [file pone.0186357.s001.zip › S1 Dataset/Fig S5A.pptx]
